# Molecular Interactions and Protein-Induced DNA Hairpin in the Transcriptional Control of Bacteriophage Ø29 DNA

**DOI:** 10.3390/ijms11125129

**Published:** 2010-12-13

**Authors:** Ana Camacho, Margarita Salas

**Affiliations:** Institute Eladio Viñuela (CSIC), Center of Molecular Biology Severo Ochoa (CSIC-UAM), Madrid Autonomous University, 28049 Madrid, Spain; E-Mail: msalas@cbm.uam.es

**Keywords:** transcription regulator, nucleoprotein complex, protein-DNA recognition, direct-indirect-readout, protein-induced DNA hairpin, A-track, minor groove malleability, arginine in minor groove

## Abstract

Studies on the regulation of phage Ø29 gene expression revealed a new mechanism to accomplish simultaneous activation and repression of transcription leading to orderly gene expression. Two phage-encoded early proteins, p4 and p6, bind synergistically to DNA, modifying the topology of the sequences encompassing early promoters A2c and A2b and late promoter A3 in a hairpin that allows the switch from early to late transcription. Protein p6 is a nucleoid-like protein that binds DNA in a non-sequence specific manner. Protein p4 is a sequence-specific DNA binding protein with multifaceted sequence-readout properties. The protein recognizes the chemical signature of only one DNA base on the inverted repeat of its target sequence through a direct-readout mechanism. In addition, p4 specific binding depends on the recognition of three A-tracts by indirect-readout mechanisms. The biological importance of those three A-tracts resides in their individual properties rather than in the global curvature that they may induce.

## 1. Introduction

Viral genes are expressed in a time-dependent manner for optimization of protein function. Gene expression is regulated primarily at the level of transcription initiation, mainly by σ factors and by transcription factors that facilitate or prevent interactions of the RNA polymerase (RNAP) with the promoter. To initiate transcription from specific promoters, the bacterial RNAP core must associate with the initiation factor σ, which contains determinants that allow sequence-specific interactions with promoter sequences [1]. A family of proteins known as “anti-σ factors” inhibits promoter utilization by targeting specific σ factors. The founding member of “anti-σ factors” is the AsiA protein of bacteriophage T4, which inhibits transcription from bacterial promoters and phage early promoters, and co-activates transcription from phage middle promoters [2–4]. Adding complexity to this regulation, anti-σ factors are regulated by anti-anti-σ factors that turn on σ factor activity, and co-anti-σ factors that act in concert with their associated anti-σ factor to inhibit or redirect σ activity [5].

Transcription factors are mostly regulatory proteins that bind to DNA sequences generally at or nearby the promoter sequence. These sequence-specific protein interactions are usually responsible for regulating transcription initiation [6]. However, some proteins that bind DNA without sequence specificity, such as the so–called “nucleoid proteins”, are also responsible for transcription regulation. Although bacteria do not have nucleosomes, they do have nucleoid proteins such as Fis or H-NS, which organize the genomes and bend DNA upon binding. Both proteins regulate transcription by affecting the DNA structure as well as antagonizing the function of other transcription factors, mainly acting as repressors [7–9]. Transcription factors were initially classified as activators or repressors if they improved or inhibited transcription, respectively. However, both activators and repressors exert dual functions depending on how and where they bind to the DNA [10–20]. The CI dimeric DNA-binding protein of phage *λ* can function as a repressor or activator, exerting the transition from one program of *λ* gene expression to another upon the formation of a higher-order protein-DNA complex [21]. Similarly, the TyrR protein of *Escherichia coli* is a dimer capable of self-association to hexamers. TyrR dimers activate transcription, but TyrR hexamers repress transcription binding to targets that overlap the promoter sequence [22]. Furthermore, most transcriptional regulatory systems rely on the function of more than one regulatory protein, where functional interaction between them results in antagonism or synergism of their functions [23–26]. Binding of regulators frequently affects locally the topology of the DNA with, in some cases, great distortion of the double-helix. Among those topological changes, DNA bending is a common feature that allows distal regulators to act synergistically allowing for correct interactions between regulators and the transcription machinery, or providing an appropriate conformation of the promoter for its interaction with the RNAP [27–33].

## 2. Molecular Requirements in the Transcriptional Switch from Early to Late Gene Expression During Bacteriophage Ø29 Infection

Phage Ø29 gene expression is directed by the *Bacillus subtilis* σ^A^-RNAP [34,35]. The core enzyme of *B. subtilis* has a subunit composition of β, β’, α_2_ and ω, homologous to the *E. coli* enzyme. The *B. subtilis* σ^A^ subunit is homologous to *E. coli* σ^70^; both recognize the same consensus sequences at the −35 and −10 hexamers [36–43].

**Figure 1.**
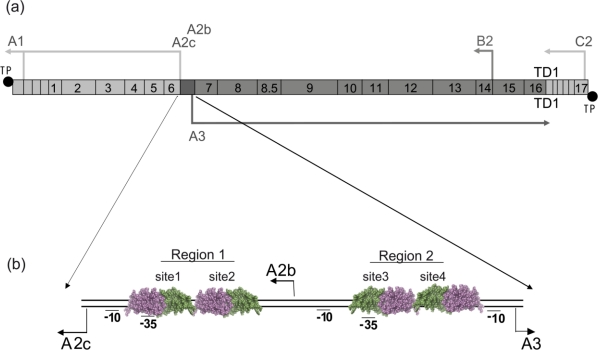
(**a**) Genetic and transcription map of phage Ø29. Genes are indicated by numbers from 1 to 17. Location of the promoters A1, A2c, A2b, A3, B2 and C2 are indicated, and the transcription terminator TD1 is denoted; (**b**) detail of the Ø29 genome intergenic region between early promoter A2c and late promoter A3. Protein p4 dimer is represented in violet-green. Protein p4 binding region 1 contains sites 1 and 2 and region 2 contains sites 3 and 4.

During Ø29 infection of *B. subtilis*, only those genes involved in DNA replication and transcription regulation are expressed at early times (Figure 1) [44]. Early genes are located at both ends of the genome and are all coded by the same DNA strand. Genes coding for structural proteins and for proteins involved in morphogenesis and cell lysis, centered in the genome, are coded in the complementary DNA strand and are transcribed at later times of infection. Transcription starting at the main early promoters, A2b and A2c, gives rise to viral proteins p6, p5, p4, p3, p2 and p1. The weak A1 promoter, located at the left end of the genome, is involved in the production of a small transcript essential in the packaging of DNA into the viral prohead [45]. Promoter B2 gives rise to short anti-sense transcripts to the late policistronic mRNA [46]. The other early promoter, C2, is located at the right end of the genome and drives the expression of genes involved in DNA replication. Late genes are expressed from a single promoter, A3. Initiation from the late A3 promoter, of low homology with the consensus sequence for σ^A^-RNA polymerase, requires early protein synthesis.

Regulation of gene expression during the development of Ø29 has proven to be a very powerful system to analyze different molecular mechanisms of transcription regulation based on formation of DNA-protein complexes and on specific DNA sequence recognition [47]. Ø29 suppressor-sensitive mutants in early genes 4 and 6 have impaired transcription when they infect a non-suppressor host [48,49]. Protein p4 binds to specific target sites and is required for the activation of the late promoter A3 and for the repression of early promoters A2b and A2c [44]. Protein p6 is a nucleoid-type protein that binds in a non-sequence specific manner to the phage DNA, generating large nucleoprotein complexes [50,51]. The p6-DNA complex formed at the right end of the genome represses promoter C2 [52,53]. In addition, p6 cooperates with p4 in transcription regulation [54]. Both proteins bind synergistically to the sequence containing early promoters A2c, A2b and late promoter A3 resulting in a multimeric complex that elicits the switch from early to late transcription by repressing early promoters A2c and A2b and simultaneously activating late promoter A3 [55].

## 3. Protein p4-DNA Complex: Direct and Indirect-Readout Mechanisms Involved in the Recognition of Target Sequences

Most DNA binding proteins recognize their targets through interactions between their amino acid side chains and DNA bases (direct-readout). However, protein-DNA complex formation frequently requires additional interactions whereby bases not contacted by the protein and apparent unspecific interactions provide specificity by an “indirect-readout” mechanism [56–59]. The affinity of a protein for its DNA target by indirect-readout relies on the fact that B-DNA exhibits a high degree of topological variation depending on its sequence. Aspects such as intrinsic curvature, topology of major or minor grooves, local geometry of backbone phosphates, flexibility, and water-mediated hydrogen bonds contribute to protein-DNA specificity [60–63].

Protein p4 is a DNA binding protein [64–66] that binds to two regions of the phage Ø29 genome encompassing the sequences from promoter A2c to promoter A3 (Figure 1) [67]. Each region contains two imperfect inverted repeats and each inverted repeat is an independent p4 binding site. Binding sites, with the consensus sequence 5′-CTTTTT-15 base pairs-AAAATG-3′, were named sites 1 to 4. Protein p4 binds two-fold more efficiently to site 3 than to site 1, and about five-fold better to site 1 than to site 2; site 4 is the lowest affinity binding site.

**Figure 2.**
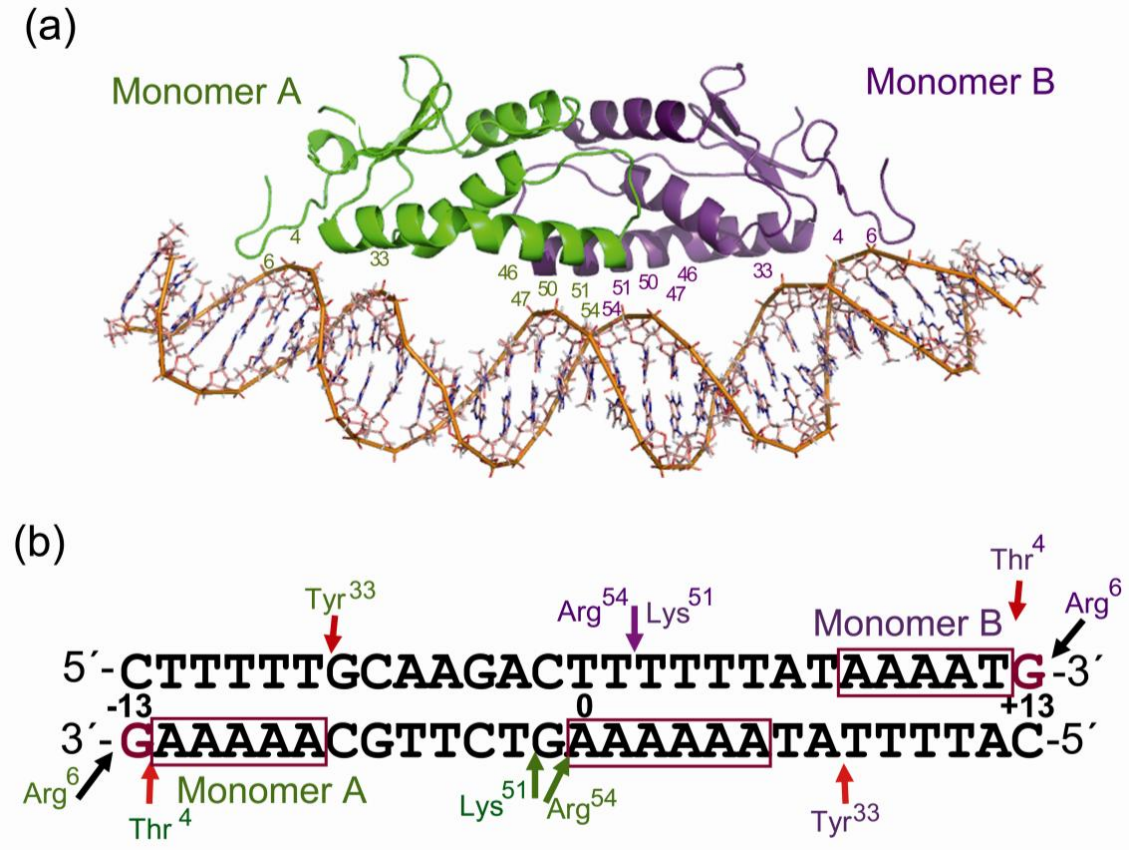
(**a**) Structure of the p4 dimer in complex with site 3. Monomers of the p4 dimer are represented in green and purple, and each monomer is distinguished as A (green) or B (purple) depending on its position with respect to the terminal repetition of site 3. Numbered are amino acids involved in DNA recognition and those of the dimerization region; (**b**) scheme of site 3 showing the protein-DNA interactions from the 3D structure [68]. Amino acid interactions with the DNA are marked with arrows.

The structure of p4 showed an elongated dimer of two identical subunits (Figure 2a) [68]. Each protomer consists of five anti-parallel β-sheets, four α-helices and one 3_10_-helix. This is a novel fold, and searching for structurally related proteins [69] revealed no relatives to p4. Another peculiarity of p4 is the structural element present at the *N*-terminus where the polypeptide chain from Pro^2^ to Gln^5^ runs anti-parallel to the stretch from Arg^6^ to Asp^11^. This structural element, named “the N-hook,” is a key feature for DNA recognition. In the structure of the p4-DNA complex, two p4 protomers are bound to the same face of the DNA helix. The DNA presents a continuous curved B conformation towards the bound protein that correlates with minor groove narrowing at the concave face and minor groove widening at the convex face, while the major groove is quite regular. The hooks*,* located at the tips of the p4 dimer, intrude into the DNA major groove making the only amino acid-base contact of the complex. The guanidinium group of Arg^6^ bonds with G at positions + and −13 (±13). In addition, three positively charged patches in the p4 dimer interact with DNA backbone phosphates at three separated A-tracts (Figure 2b) [68]. Two of those A-tracts are externally located and placed in the inverted repeats of the target; the third is near position 0. In the external A-tracts, Thr^4^ contacts the phosphate of the base at position ±12. In addition, helix α1 residue Tyr^33^ contacts DNA phosphates at position ±8 on the opposite DNA strand. Residues Lys^51^ and Arg^54^ asymmetrically contact phosphates at the central minor groove [68]. Analysis of alanine-substituted proteins at those residues, as well as the study of p4 interaction with mutated binding sites, provided important insights into the determinants required for p4-DNA complex formation. Alanine substitution of Arg^6^, the amino acid responsible for base recognition, or substitution of G ±13 were deleterious for p4 binding. Substitution of Thr^4^ or Tyr^33^ by alanine or disruption of the two external A-tracts by replacement of the A•T base pairs at position ±10 or ±11 for the less deformable base pair C•G, which increases the energy required to distort the DNA, abolished p4 binding [70]. Consequently, the N-hook motif is a new protein sub-structure for DNA binding. The motif establishes proper recognition of the DNA sequence by a direct-readout mechanism that involves Arg^6^-G ±13 interaction and with Thr^4^ contributing to the indirect-readout mechanism of recognition of the external A-tracts.

We generated Molecular Dynamics (MD) simulations of DNA and p4-DNA complexes to investigate the basis for the p4-DNA complex specificity [70]. In the absence of p4, the free DNA sequence corresponding to site 3 relaxes to a non-bent B conformation. Hence, the bent structure of the DNA in the p4-DNA complex is a consequence of the induced conformational modification impressed by p4. In agreement, p4 does not require intrinsically bent DNA for binding [67]. Despite the two-fold symmetry of the protein dimer, the target DNA has pseudo-inverted repeats (Figure 2b). One protomer (monomer A) interacts at the end containing the sequence 5′-AAAAAG-3′, and the other (monomer B) at the opposite end at the sequence 5′-AAAATG-3′. Studies on the functionality of this asymmetry led us to conclude than each inverted repeat contributes to p4 binding affinity differently since the monomers display dissimilar binding entropies; monomer B presents higher entropic stability than monomer A [70]. This is probably due to the fact that the pyrimidine-purine T/G step is more susceptible to deformation than the A/G step due to its smaller area of base overlap that will allow a better orientation of G + 13 for its interaction with Arg^6^ (Figure 2). Therefore, the sequence-dependent characteristics of the external A-tracts provide an indirect-readout of the sequence by affecting the optimal complementarities, both for amino acid-base hydrogen bonding and to favor interactions between amino acids and phosphates on the narrowed minor groove.

**Figure 3.**
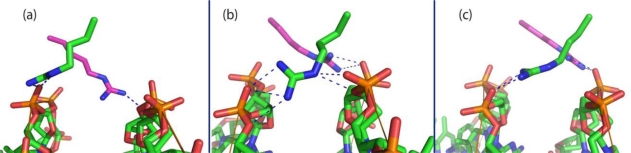
Selected images of Arg^54^ rearrangement at (**a**) 0 ns, (**b**) 2.4 ns and (**c**) 4 ns. Arginine^54^-monomer B (violet) interacts only with T + 2 phosphate along the trajectory. Arginine^54^-monomer A (green) interacts with G − 1 phosphate in (**a**); with G − 1 and T + 3 phosphates and with deoxyribose O3 atoms of A0 and T + 3 in (**b**) and with G − 1 phosphate in (**c**).

The *C*-termini of α1 helices contain a cluster of 12 positively charged amino acids, located after a kink that maintain the helix almost parallel to the DNA axis (Figure 2). From these amino acids only Lys^51^ and Arg^54^ contact the DNA backbone in the p4-DNA structure and do so at the central A-track, although not symmetrically (Figure 2(b)). One monomer establishes salt bridges with T − 2 and G − 1 phosphates, and the other monomer contacts the T + 2 phosphate across the minor groove [68]. Studies on the contribution of those amino acids to p4-DNA complex stabilization and sequence specific recognition were carried out by analyzing punctual mutated proteins, mutated DNA targets and by MD simulations. The results showed that A·T pairs from position 0 to +2 and Arg^54^ are critical for p4-specific binding [71]. It is remarkable that while the location of Arg^54^-monomer B was quite stable along the MD simulation, interacting mainly with the T + 2 phosphate, the residue of monomer A modified greatly its position in the p4-DNA complex (Figure 3). The residue, originally superficially positioned across the minor groove contacting A0 and G − 1 phosphates, moves into the groove between residues T + 3 and G − 1. The movement seems to be accomplished by the establishment of simultaneous hydrogen bonds at both DNA strands with the G − 1, T + 2 and T + 3 phosphates and with the deoxyribose O3 atoms of A0 and T + 1. Arg^54^-monomer A, stapling both DNA strands, would narrow the minor groove locally. Hence, despite being chemically equivalent and in identical monomers, the arginines differed in their interactions with DNA. Participation of arginines in DNA interaction has been the subject of a number of investigations. The arrangement of Arg^54^ in the p4-DNA complex differs from that in the complexes of Hox, histones or phage-434 repressor [72–74]. In the nucleosomes, the arginines are positioned asymmetrically in the minor groove frequently bridging O4 atoms of nucleotides i and i + 3 [75]. In the 434 repressor-DNA complex, the arginine is docked in the centre of the groove with the guanidium group bridging the deoxyribose O4 atoms from nucleotide i and i + 2. In the p4-DNA complex Arg^54^ generally bridged deoxyribose O3 atoms with phosphates. Therefore, p4 uses its Arg^54^ and the inherent properties of the central A-tract in order to create specific target recognition.

Binding of transcriptional regulators to specific sequences must be strong enough to allow the regulatory protein to bind to its target site in the presence of competing non-specific binding, but not so tight as to impede the normal turnover of the regulatory processes. This scenario would be archived if the specific DNA-protein interactions utilize an assortment of direct- and indirect-readout mechanisms. Direct-readout mechanisms implying several amino acid-base interactions confer higher specificity but may produce too tight interaction. However, a direct-readout mechanism based on a single amino acid-base interaction, as it occurs with p4, complemented with several indirect-readout mechanisms would produce the grade of specificity and stability required for appropriate turnover. Moreover, the indirect-readout mechanisms described here could enable binding of proteins with negligible direct-readout recognition such as p4, histones and some transcriptional regulators to use information in the minor groove to achieve the required grade of DNA-binding specificity.

## 4. Zipper Model for p4 Specific Sequence Recognition and DNA Binding

In the p4-DNA complex, stability is a consequence of p4-induced conformational modification of the DNA, whereas the primary function of the DNA is its ability to acquire a conformation capable of enhancing positive interactions with p4. Taking into account that: (i) the asymmetry of the DNA target is functionally required for p4-DNA interaction; (ii) p4 curves the DNA, and (iii) the distance from G − 13 to G + 13 is about 90 Å while the 75 Å distance from the Arg^6^ of one of the monomers to the Arg^6^ of the other monomer is too short for simultaneous interaction of both monomers, we propose a zipper binding model for p4. In the model, one of the p4 monomers interacts first with the higher entropic stability inverted repeat sequence, 5′-AAAATG-3′. The N-hook gets introduced into the major groove, providing the Arg^6^-G + 13 specific interaction. Subsequent local narrowing of the proximal minor groove mediated by the contacts of Thr^4^ and Tyr^33^ at both edges of the nearest A-track will approach the central minor groove to the patch of positive amino acids. Here, Arg^54^-DNA interactions would narrow the groove. Two consecutive minor grooves narrowed in the same direction will progressively bend the DNA allowing the 5′-AAAAAG-3′ inverted repeat to reach the hook of monomer A [47].

## 5. Regulation of the Switch from Early to Late Gene Expression

**Figure 4.**
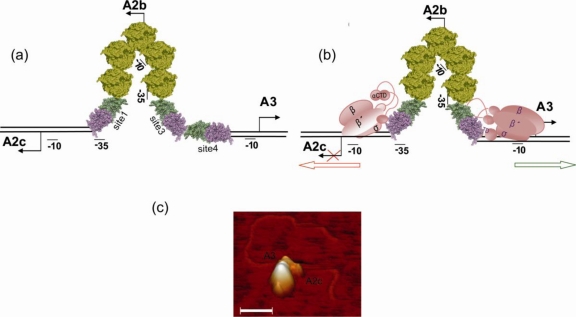
Model and Atomic Force Microscopy (AFM) images representing the hairpin on the p4-p6-DNA and on the p4-p6-RNAP-DNA complexes. (**a**) Localization of proteins and topology of the DNA on the ternary p4-p6-DNA complex. Protein p4 (dimer in violet-green) and protein p6 (dimer in yellow) binding between promoters A2c and A3 results in the formation of the nucleoprotein-hairpin. P4 bound to sites 1 and 3 partially overlay the −35 elements of early promoters A2b and A2c, respectively; (**b**) Model of the p4-p6-RNAP-DNA quaternary complex. The RNAP is represented stably bound to promoter A3 but in an unstable interaction with promoter A2c; (**c**) AFM image of a quaternary complex p4-p6-DNA-RNAP. The image shows the large volume of the RNA polymerase bound to promoter A3 and to its right the hairpin structure originated by the binding of p4 and p6 to the DNA.

Upon Ø29 infection of *B. subtilis*, the host RNAP starts transcription from early promoters A2b and A2c. RNAP recognizes promoters A2c and A2b through interaction of the α and σ subunits at the promoter elements [76]. Efficient stabilization of the closed complex at late promoter A3 requires protein p4 since the consensus of its −35 element is poor [77]. Synthesis of early mRNA gives rise to the production of proteins p4 and p6. P4 binding sites 1 to 4 are placed between promoters A2c and A3 with sites 1 and 3 overlapping the −35 element of promoters A2c and A2b, respectively (Figure 4). When p4 is bound to its four binding sites, the two DNA strands build an angle around p4 resulting in a 13 nm hairpin structure [78]. This hairpin might be the triggering factor for the preferential binding of p6 between p4 sites 1 and 3 (displacing p4 from site 2) that leads to the stabilization of the nucleoprotein-hairpin that modifies the activity of promoters A2c, A2b and A3 [76]. The nucleoprotein-hairpin allows RNAP recognition of promoter A2c giving rise to a closed complex with impeded isomerization to the open complex. No transcription complex is detected at promoter A2b, most probably due to the topological modification of the promoter sequence located at the apex of the nucleoprotein-hairpin. On the other hand, the p4 dimer at site 3, further stabilized by p6, interacts with RNAP overcoming the rate-limiting step (closed-complex formation) of promoter A3 [79,80].

## 6. Conclusions

The switch from early to late transcription of the Ø29 genome is tightly regulated to ensure the appropriate sequence of gene expression. Repression of early promoters A2c and A2b and activation of the late promoter A3 are simultaneously regulated in a sophisticated manner by proteins p4 and p6, where protein p4 has a leading role in the process.

The study of protein p4 revealed novel protein-DNA interaction paradigms: (i) The p4 structure adds a new DNA binding motif to the catalogue of DNA binding protein motifs, the N-hook; (ii) p4 recognizes its targets through direct-readout of the boundary guanines in its target sites and by two additional indirect-readout mechanisms. Both indirect-readout mechanisms are based on the malleability of A-tracts. First, remodeling the topology of the external A-tracks, p4 provides a better adjustment of the N-hook to the DNA. Second, the over-winding of the central minor groove by the insertion of Arg^54^ would narrow it providing optimal complementarity between one p4 surface and its target. Therefore, p4 creates specificity in the protein-DNA complex using the intrinsic properties of minor groove A-tracts.

Protein p4 bound to its targets remodel 120 base pairs of DNA to the structure of a nucleoprotein-hairpin that is stabilized by the incorporation of p6. The nucleoprotein-hairpin is the key factor that coordinates gene expression since the switch from early to late transcription is the interplay between the RNAP and the p4-p6 complex for binding to the sequence containing promoters A2c, A2b and A3. Efficient promoter complex formation and transcription initiation requires the appropriate positioning of the RNAP at the promoter. The stability of the hairpin structure, which depends on the availability of proteins p4 and p6 in the cell, might be critical. The hairpin impairs the correct interaction of the RNAP at early promoters A2c and A2b and simultaneously activates late promoter A3 stabilising the primary transcriptional complexes.
